# Helical filament structure of the DREP3 CIDE domain reveals a unified mechanism of CIDE-domain assembly

**DOI:** 10.1107/S2059798321010767

**Published:** 2021-11-11

**Authors:** So Yeon Lee, Sunghark Kwon, Hyun Ji Ha, Sung Hoon Lee, Hyun Ho Park

**Affiliations:** a College of Pharmacy, Chung-Ang University, Seoul 06974, Republic of Korea; b Department of Global Innovative Drugs, Graduate School of Chung-Ang University, Seoul 06974, Republic of Korea; c Department of Biotechnology, Konkuk University, Chungju, Chungbuk 27478, Republic of Korea

**Keywords:** apoptotic DNA fragmentation, CIDE family, crystal structure, DREP3, higher-order structure

## Abstract

The cell-death-inducing DFF45-like effector (CIDE) domain is a protein-interaction module comprising ∼80 amino acids and was initially identified in several apoptotic nucleases and their regulators. CIDE-domain-containing proteins were subsequently identified among proteins involved in lipid metabolism. Given the involvement of CIDE-domain-containing proteins in cell death and lipid homeostasis, their structure and function have been intensively studied. Here, the head-to-tail helical filament structure of the CIDE domain of DNA fragmentation factor-related protein 3 (DREP3) is presented. The helical filament structure was formed by opposing positively and negatively charged interfaces of the domain and was assembled depending on protein and salt concentrations. Although conserved filament structures are observed in CIDE family members, the structure elucidated in this study and its comparison with previous structures indicated that the size and the number of molecules used in one turn vary. These findings suggest that this charged-surface-based head-to-tail helical filament structure represents a unified mechanism of CIDE-domain assembly and provides insight into the function of various forms of the filament structure of the CIDE domain in higher-order assembly for apoptotic DNA fragmentation and control of lipid-droplet size.

## Abstract 2

The CIDE domain was initially identified in apoptotic nucleases and now forms a highly conserved family with diverse functions ranging from cell death to lipid metabolism. Based on structural determination of the DREP3 domain, it is suggested that the head-to-tail helical filament structure might be a unified mechanism of CIDE-domain assembly and represents a critical higher-order scaffolding structure that is important for the function of CIDE-domain-containing proteins in DNA fragmentation and lipid-droplet fusion.

## 1. Introduction

DNA fragmentation into a laddered pattern comprising multiples of 180–200 base-pair (bp) fragments is a biochemical hallmark of apoptotic cells (Nagata, 2000 ▸; Batistatou & Greene, 1993 ▸; Enari *et al.*, 1998 ▸). The main player in apoptotic DNA fragmentation is nuclease DNA-fragmentation factor of 40 kDa (DFF40), the nuclease activity of which is inhibited by DFF45 via the formation of a heterodimeric complex (Liu *et al.*, 1997 ▸; Enari *et al.*, 1998 ▸). Upon triggering apoptotic signalling, DFF45 is cleaved by activated caspases to release DFF40 (Liu *et al.*, 1999 ▸; Thomas *et al.*, 2000 ▸). The dissociated DFF40 translocates to the nucleus and digests genomic DNA, producing 180–200 bp DNA fragments (Sakahira *et al.*, 1999 ▸; Liu *et al.*, 1997 ▸). A heterodimeric complex of caspase-activated deoxyribonuclease (CAD, a DFF40 homologue) and its inhibitor (ICAD, a DFF45 homologue) was identified in a mouse-based study (Sakahira *et al.*, 1998 ▸), which showed the same activity and control mechanism as observed in humans.

Human DFF40 and DFF45 (and mouse CAD and ICAD) contain a conserved domain at the N-terminus referred to as the cell death-inducing DFF45-like effector (CIDE) domain (Inohara *et al.*, 1998 ▸), which mediates heterodimer formation. In *Drosophila*, four CIDE-domain-containing DFF-related proteins (DREP1–DREP4) have been identified by genome-sequence analysis (Mukae *et al.*, 2000 ▸; Inohara & Nuñez, 1999 ▸; Yokoyama *et al.*, 2000 ▸). The biochemical characterization of CIDE–CIDE interactions in DREP1–DREP4 showed that the DREP2 and DREP4 nucleases interact with and are inhibited by DREP1 and DREP3 (Lee & Park, 2014 ▸; Park & Park, 2012 ▸, 2013 ▸). A previous study reported that DREP2 acts as a synaptic protein and exhibits a unique function in learning and behavioural adaptation (Andlauer *et al.*, 2014 ▸). Another family of CIDE-containing proteins includes CIDE-A, CIDE-B and FSP27 (also known as CIDE-C), which play roles in lipid metabolism at the lipid-droplet level rather than in apoptotic DNA fragmentation (Xu *et al.*, 2012 ▸; Traini & Jessup, 2009 ▸; Yonezawa *et al.*, 2011 ▸). Functional failure of CIDE-domain-containing proteins has been linked to obesity, diabetes, liver steatosis and cardiovascular diseases (Xu *et al.*, 2012 ▸).

A structural study of CIDE domains identified an α/β-roll structure comprising two α-helices and five β-strands (Lugovskoy *et al.*, 1999 ▸). Charge-complementary-based heterodimerization and homodimerization of CIDE domains have also been revealed by structural studies of the DFF4–DFF45 (CAD–ICAD) complex and FSP27, respectively (Zhou *et al.*, 2001 ▸; Lee *et al.*, 2013 ▸). Interestingly, recent structural studies of CIDE domains in DREP4 and DREP2 and a study of the DFF40–DFF45 system indicated that CIDE domains form an open-ended helical filament, which is an important scaffolding structure required for the execution of their roles in apoptotic DNA fragmentation (Choi *et al.*, 2017 ▸; Ha & Park, 2018 ▸).

In this study, we present the crystal structure of the DREP3 CIDE domain. Structural and mutational analyses and biochemical studies revealed that the head-to-tail helical filament structure may be a unified mechanism of CIDE-domain assembly, which represents a critical higher-order scaffolding structure important for the function of CIDE-domain-containing proteins in DNA fragmentation and lipid-droplet fusion. Additionally, we revealed that the helical filament structures are diverse in length, diameter and helical properties.

## 2. Materials and methods

### 2.1. Sequence alignment

The amino-acid sequences of CIDE domains were analysed using *Clustal Omega* (http://www.ebi.ac.uk/Tools/msa/clustalo/).

### 2.2. Protein expression and purification

The expression plasmid for the DREP3 CIDE domain (corresponding to residues Phe112–Asp195) was constructed by inserting the amplicon, digested with NdeI and XhoI, into a pET-21a vector. The gene sequence information was derived from UniProt (ID Q0E9B7; https://www.uniprot.org). The expression vector was delivered into *Escherichia coli* BL21 (DE3) cells using heat shock at 42°C, and the transformed bacteria were spread on a lysogeny broth (LB) agar plate containing ampicillin and incubated at 37°C for 16 h. A single recombinant colony was selected and cultured overnight at 37°C in 5 ml LB containing 50 µg ml^−1^ ampicillin, after which the cells were transferred to a large-scale culture in 1 l medium. Upon reaching an optical density at 600 nm of ∼0.6, 0.5 m*M* isopropyl β-d-1-thiogalactopyranoside (IPTG) was added to the culture to induce gene expression. IPTG-treated cells were chilled on ice and further cultured at 20°C for 18 h in a shaking incubator. Subsequently, the bacterial cells were harvested and the pellet was resuspended in 20 ml lysis buffer (20 m*M* Tris–HCl pH 7.9, 500 m*M* NaCl, 25 m*M* imidazole). After adding phenylmethanesulfonyl fluoride (Sigma–Aldrich, St Louis, USA), the cells were disrupted by sonication on ice with six bursts of 30 s each with a 60 s interval between each burst. The cell lysate was centrifuged at 10 000*g* and 4°C for 30 min to remove cell debris, and the supernatant was collected and mixed overnight with nickel–nitrilotriacetic acid (Ni–NTA) resin (Qiagen, Hilden, Germany) by gentle agitation at 4°C. The resulting mixture was loaded onto a gravity-flow column. The Ni–NTA resin in the column was washed with 50 ml wash buffer (20 m*M* Tris–HCl pH 7.9, 500 m*M* NaCl, 60 m*M* imidazole) to remove unbound proteins. For elution, 0.5 ml elution buffer (20 m*M* Tris–HCl pH 7.9, 500 m*M* NaCl, 250 m*M* imidazole) was added to the column five times. The resulting eluate was concentrated to 30 mg ml^−1^ and subjected to size-exclusion chromatography (SEC) using an ÄKTAexplorer system (GE Healthcare, Chicago, Illinois, USA) equipped with a Superdex 200 Increase 10/300 GL 24 ml column (GE Healthcare) pre-equilibrated with SEC buffer (20 m*M* Tris–HCl pH 8.0, 500 m*M* NaCl). The SEC peak fractions were pooled, concentrated to 9 mg ml^−1^, flash-frozen in liquid nitrogen and stored at −80°C until further use. Protein purity was assessed using SDS–PAGE.

Site-directed mutagenesis was performed using a QuikChange kit (Stratagene, San Diego, California, USA) according to the manufacturer’s instructions. Mutagenesis was confirmed by sequencing, and the mutant proteins were expressed and purified using the methods described above.

### 2.3. Crystallization and data collection

Crystals of the DREP3 CIDE domain were initially obtained by the hanging-drop vapour-diffusion method at 20°C. Briefly, 1 µl 11 mg ml^−1^ protein sample in buffer (20 m*M* Tris–HCl pH 8.0, 500 m*M* NaCl) was mixed with an equal volume of reservoir solution (0.1 *M* citric acid pH 4.0, 0.8 *M* ammonium sulfate) and the droplet was allowed to equilibrate against 400 µl mother liquor. The crystallization conditions were further optimized and finally adjusted to obtain a buffer composition comprising 0.1 *M* citric acid pH 3.4, 0.6 *M* ammonium sulfate. Diffraction-quality crystals appeared within three days and grew to a maximum size of 0.3 × 0.1 × 0.1 mm. For data collection, crystals were soaked in mother liquor supplemented with 40%(*v*/*v*) glycerol as a cryoprotectant, mounted and flash-cooled in a nitrogen stream at −178°C. Diffraction data were collected on beamline 5C at the Pohang Accelerator Laboratory (PAL), Pohang, Republic of Korea at a wavelength of 1.0000 Å. The diffraction data were indexed, integrated and scaled using *HKL*-2000 (Otwinowski & Minor, 1997 ▸).

### 2.4. Structure determination and analysis

The structure of the DREP3 CIDE domain was determined by molecular replacement performed using *Phaser* (McCoy *et al.*, 2007 ▸). The monomeric structure of the DREP2 CIDE domain (PDB entry 4d2k; Choi *et al.*, 2017 ▸), which has 28% amino-acid sequence identity to the DREP3 CIDE domain, was used as the search model. The initial model was built automatically with *AutoBuild* in *Phenix* and was completed with *Coot* (Liebschner *et al.*, 2019 ▸; Emsley *et al.*, 2010 ▸). Model refinement was iteratively performed using *phenix.refine* in *Phenix* (Liebschner *et al.*, 2019 ▸), and the quality of the model was validated using *MolProbity* (Chen *et al.*, 2010 ▸). All structural figures were generated using *PyMOL* (DeLano & Lam, 2005 ▸).

### 2.5. Electron microscopy (EM)

DREP3 protein samples after Ni–NTA batch purification were diluted to 0.2 mg ml^−1^. For negative staining, 10 µl protein sample was placed onto a glow-discharged copper grid, stained with 1% uranyl formate pH 4.5 for 20 s and air-dried. The grid was imaged using a Tecnai G^2^ Spirit BioTWIN transmission electron microscope and was recorded using an AMT 2k CCD camera at the Chung-Ang University Electron Microscopy Facility, Seoul, Republic of Korea.

### 2.6. SEC–multiangle light scattering (SEC-MALS) analysis

The absolute molar mass was determined using SEC-MALS. The purified target protein was passed through a 0.2 µm syringe filter and loaded onto a Superdex 200 10/300 gel-filtration column (GE Healthcare) pre-equilibrated with SEC buffer. The mobile-phase buffer flowed at a rate of 0.4 ml min^−1^ at 25°C. A DAWN TREOS MALS detector (Wyatt Technology, Santa Barbara, California, USA) was interconnected with the ÄKTAexplorer system (GE Healthcare). The molecular mass of bovine serum albumin was used as the reference value. The absolute molecular mass was assessed using *ASTRA* (Wyatt Technology). Protein samples at a concentration of 10 mg ml^−1^ were used in the MALS experiments.

### 2.7. Circular-dichroism (CD) spectroscopy

The secondary structures were measured by CD spectroscopy using a J-715 spectropolarimeter at the Korea Basic Science Institute, Daejeon, Republic of Korea. Far-ultraviolet (UV) CD spectra were recorded from 200 to 260 nm at 25°C in a quartz cuvette with a 0.1 cm path length using a bandwidth of 1.0 nm, a speed of 50 mm min^−1^ and a 5 s response time. The protein samples were diluted to 0.1 mg ml^−1^ in buffer consisting of 20 m*M* Tris–HCl pH 8.0, 500 m*M* NaCl before use. Four scans were accumulated and averaged.

### 2.8. Accession number

Atomic coordinates and structural factors have been deposited in the Protein Data Bank as PDB entry 7v6e.

## 3. Results

### 3.1. Structure of the DREP3 CIDE domain

**Figure 1:**
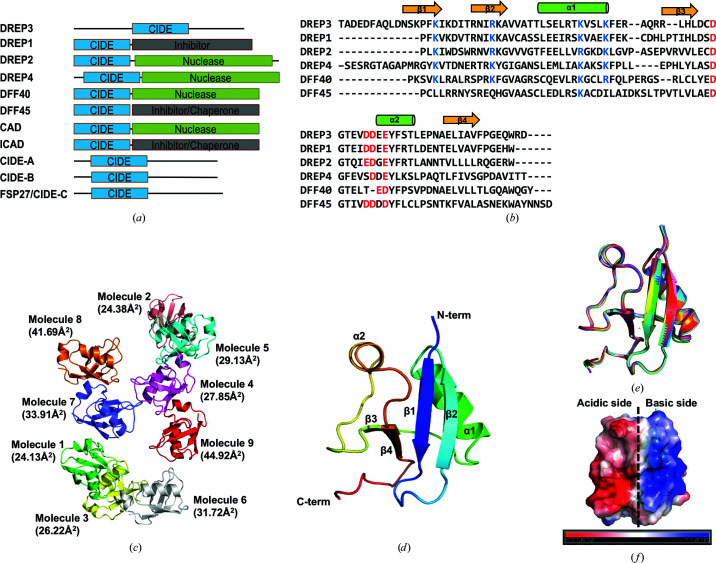
Structure of the cell-death-inducing DFF45-like effector (CIDE) domain in DREP3. (*a*) Domain organization of CIDE-domain-containing proteins. The location of the CIDE domain is shown. (*b*) Sequence alignment of various CIDE domains. Previously identified conserved residues involved in hetero-oligomeric and homo-oligomeric complexes are coloured blue for basic residues and red for acidic residues. Secondary structures including α-helices and β-sheets are labelled. DFF40 and DFF45 are from *Homo sapiens*, while DREP1–DREP4 are from *Drosophila melanogaster*. (*c*) Ribbon diagram of the nine molecules of the DREP3 CIDE domain present in the asymmetric unit. The nine molecules are shown separately and are numbered from 1 to 9 according to their average temperature factor. Values in parentheses indicate the average temperature factors. (*d*) Ribbon diagram of the monomeric structure of the DREP3 CIDE domain. The chain from the N-terminus (N-term) to the C-terminus (C-term) is coloured in a spectrum from blue to red. Helices and sheets are labelled. (*e*) Structural comparion of all nine molecules detected in the asymmetric unit. The same colour code as used in (*c*) was used to indicate the superposed molecules. (*f*) Surface view of the monomeric DREP3 CIDE domain with displayed electrostatics. This is shown in the same view as in (*d*).

The CIDE domain is a conserved protein-interaction module found in several proteins involved in cell death and lipid metabolism (Fig. 1 ▸ *a*). CIDE-domain-mediated DFF40 and DFF45 interactions are important for regulating the nuclease activity of DFF40 during apoptotic DNA fragmentation. The CIDE domain forms a head-to-tail helical filament in solution, the implications of which remain undetermined, although a process related to increased local concentration through filament formation is suspected (Choi *et al.*, 2017 ▸). DREP3 is a CIDE-domain-containing protein in *Drosophila* and has been suggested to be a regulator of nucleases (Park & Park, 2012 ▸). Unlike proteins harbouring the CIDE domain at the N-terminus, the DREP3 CIDE domain is located in the middle of the protein (Fig. 1 ▸ *a*). Despite this difference, the DREP3 CIDE domain contains two conserved differently charged regions that are critical for hetero-oligomerization and homo-oligomerization (Fig. 1 ▸ *b*).

To further understand this dynamic protein-interaction module, we obtained a crystal of the DREP3 CIDE domain which had a calculated Matthews coefficient (*V*_M_) of 2.76 Å^3^ Da^−1^, corresponding to a solvent content of 55.43%, and solved the structure at a resolution of 3.0 Å. Diffraction data and refinement statistics are summarized in Table 1 ▸. The structure of the DREP3 CIDE domain was solved by molecular replacement using *Phaser* (McCoy *et al.*, 2007 ▸) with the default setup values. By using the monomeric DREP2 CIDE-domain structure (PDB entry 4d2k; Choi *et al.*, 2017 ▸) as the search model, *Phaser* found all nine molecules in the crystallographic asymmetric unit (Fig. 1 ▸ *c*). The final model contained residues 117–193 for all nine molecules. The five amino-acid residues at the N-terminus (residues 112–116) and two residues at the C-terminus (residues 194 and 195) were not included in the final model due to untraceable electron density in these regions.

The structure of the DREP3 CIDE domain exhibited the canonical CIDE-domain fold, which comprises an α/β-roll fold with two α-helices and four β-strands (Fig. 1 ▸ *d*). The two helices (α1 and α2) included residues Ile139–Lys149 and Glu169–Thr174, respectively. The four strands comprising residues Lys119–Lys124, Arg131–Ala136, Arg156–Leu159 and Glu180–Val184 are indicated as β1, β2, β3 and β4, respectively (Fig. 1 ▸ *d*). The structure of all nine molecules was nearly identical, exhibiting a root-mean-square deviation (r.m.s.d.) of around 0.3–0.4 Å when they were superimposed (Fig. 1 ▸ *e*). As a common feature, the surface of the DREP3 CIDE domain was also divided into two distinctly differently charged sides (acidic and basic; Park, 2015 ▸; Fig. 1 ▸ *f*).

**Figure 2:**
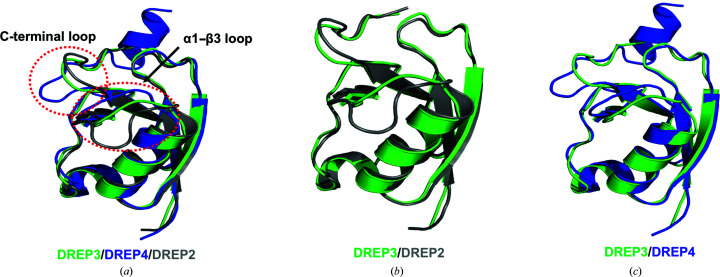
Structural comparison of the DREP3 CIDE domain with the DREP2 and DREP4 CIDE domains. (*a*) Structural alignment of a monomeric DREP3 CIDE domain with those of DREP2 (grey) and DREP4 (blue). Red dotted circles indicate the structurally different regions. (*b*, *c*) Pairwise structural alignment of the DREP3 CIDE domain with those of DREP2 and DREP4.

The newly solved structure of the DREP3 CIDE domain was compared with those of structural homologues, including DREP2 and DREP4. Molecule 1 among the nine molecules, which has the lowest temperature factor, was picked and compared by structural superposition; this indicated that the overall structures were nearly identical, as indicated by superimpositions with r.m.s.d.s of 0.7 Å (DREP2 CIDE domain) and 0.9 Å (DREP4 CIDE domain) (Fig. 2 ▸ *a*). However, the structures of the α1–β3 loop and the C-terminal loop differed (Figs. 2 ▸ *a*–2 ▸ *c*).

### 3.2. Dynamic properties of the DREP3 CIDE domain: variable homo-oligomeric forms

**Figure 3:**
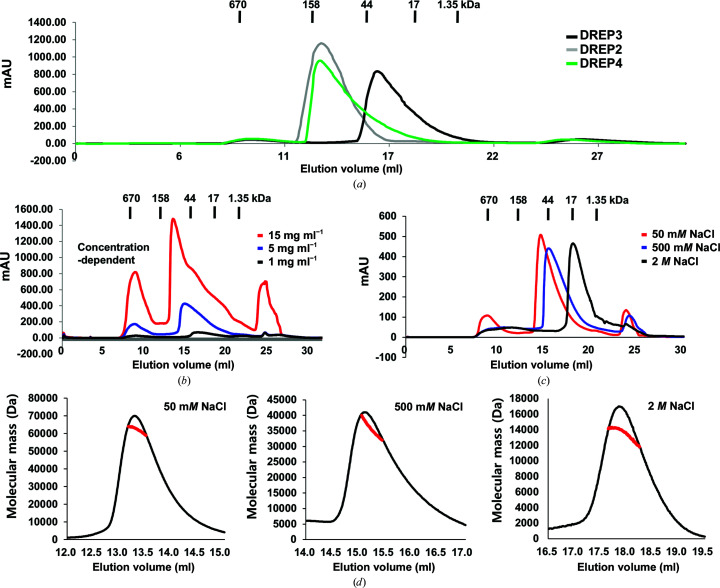
Dynamic properties of the DREP3 CIDE domain. (*a*) SEC chromatograms of the DREP3 CIDE domain and other CIDE domains. All SEC experiments were performed using 20 m*M* Tris–HCl pH 7.9, 500 m*M* NaCl buffer. (*b*) SEC chromatograms of samples of the DREP3 CIDE domain at three different concentrations. (*c*) SEC chromatograms of samples of the DREP3 CIDE domain at four different NaCl concentrations. The DREP3 CIDE domain formed large oligomers under low-salt conditions (50 m*M*) and migrated at smaller sizes with increasing salt concentration. (*d*) SEC-MALS measurements of various forms of the DREP3 CIDE domain in different concentrations of NaCl. The red line indicates the experimentally calculated molecular mass.

The dynamic assembly/disassembly property of the CIDE domain in solution is its primary feature (Lee & Park, 2014 ▸). The DREP3 CIDE domain eluted at ∼16–17 ml in SEC (in 500 m*M* NaCl), corresponding to a molecular weight of 30–40 kDa and indicating that this domain also forms a homo-oligomer in solution. Compared with the CIDE domains of DREP2 and DREP4, which form higher oligomeric complexes and elute at 12 and 13 ml by SEC (in 500 m*M* NaCl), the DREP3 CIDE domain formed a smaller homo-oligomeric complex (Fig. 3 ▸ *a*). To analyse the dynamic assembly/disassembly of the DREP3 CIDE domain, we determined its concentration-dependent self-assembly using SEC. Three independent SEC experiments performed with protein samples at three different concentrations (1, 5 and 15 mg ml^−1^) showed that larger amounts of the DREP3 CIDE domain produced larger-sized particles that eluted earlier on SEC, indicating that homo-oligomerization of the DREP3 CIDE domain is dependent on the protein concentration (Fig. 3 ▸ *b*). The void peak contained an aggregated sample of the DREP3 CIDE domain and the peak after 24 ml contained various protein aggregates that were produced during the concentration of DREP3 (Figs. 3 ▸ *b* and 3 ▸ *c*). Additionally, we assessed the salt-dependence of CIDE-domain self-assembly using SEC. As the NaCl concentration was increased the DREP3 CIDE domain eluted later in SEC, indicating that a higher oligomeric complex was dissociated in high-salt conditions. This experiment indicated that self-assembly of the DREP3 CIDE domain was also highly dependent on salt concentration (Fig. 3 ▸ *c*). The accurate molecular mass of various forms of the DREP3 CIDE domain in different concentrations of NaCl was analysed by SEC-MALS measurements, which can calculate the absolute molecular mass of a particle in solution. This experiment revealed that the molecular masses of the DREP3 CIDE domain in 50 m*M*, 500 m*M* and 2 *M* NaCl were 62.14 kDa (1.7% fitting error), 35.46 kDa (2.2% fitting error) and 13.47 kDa (1.3% fitting error), respectively, indicating that the DREP3 CIDE domain forms a higher oligomeric complex in low-salt conditions and this oligomeric complex is dissociated on increasing the salt concentration (Fig. 3 ▸ *d*).

### 3.3. The helical filament structure of the DREP3 CIDE domain

**Figure 4:**
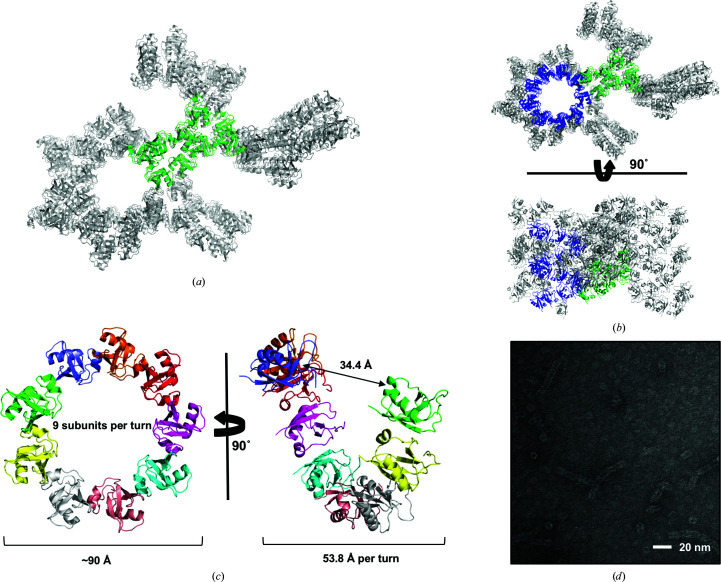
Helical assembly of DREP3 CIDE domains. (*a*) The packing arrangement in the crystal lattice. The green molecules are the nine molecules found in the asymmetric unit during structural determination. The grey molecules are symmetric molecules that packed into the crystal lattice. (*b*) Crystal-packing analysis. The blue molecules are helical molecules found in the packing arrangement. The green and grey molecules are the same molecules as described in (*a*). (*c*) The helical filament structure of the DREP3 CIDE domain constructed from symmetric molecules. Each molecule is shown in a different colour. (*d*) EM images of negative-stained DREP3 CIDE domain samples showing ring and filament structures.

As found in other CIDE domains, the surfaces of the DREP3 CIDE domain are divided into two oppositely charged surfaces, which are critical for their homo-oligomerization. Additionally, their dynamic assembly/disassembly properties (higher homo-oligomeric assembly depending on the local concentration of protein and the salt concentration) in solution were similar to those of the previously studied CIDE domains of DREP4 and DFF40 (Choi *et al.*, 2017 ▸). Based on these common properties, we hypothesized that this domain might also form a filament-like structure as previously identified in structural studies of the DREP2 and DREP4 CIDE domains (Choi *et al.*, 2017 ▸; Ha & Park, 2018 ▸). Specifically, we analysed any tentative helical structure in the DREP3 CIDE domain by investigating crystallographic packing and symmetry-related molecules (Figs. 4 ▸ *a* and 4 ▸ *b*). We observed that all nine helix-related molecules were arranged into a helical structure in the crystal, with nine molecules per turn, a rise of 53.8 Å and a diameter of ∼90 Å (Figs. 4 ▸ *b* and 4 ▸ *c*). In the crystal lattice, the helical structure was continuous and stacked along the *a* axis of the unit cell (Fig. 4 ▸ *b*).

To confirm the helical assembly of the DREP3 CIDE domain in solution and corroborate the crystal structure, we performed EM experiments. The protein sample eluted at 16–17 ml from a Superdex 200 gel-filtration column under salt conditions of 500 m*M* NaCl was used in EM experiments. EM of negatively stained samples showed that the DREP3 CIDE domain formed rings and filament-like structures with comparable diameters to those observed in the crystal structure (Fig. 4 ▸ *b*). The protein sample eluted at the void volume in SEC was aggregated as judged by EM.

### 3.4. Unified mechanism of CIDE-domain helical assembly with variable forms

**Figure 5:**
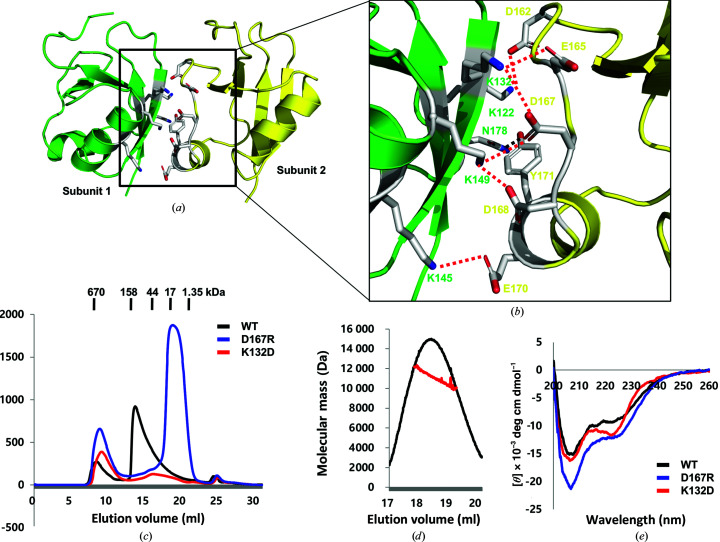
Interface analysis of the helical filament structure of the DREP3 CIDE domain. (*a*) Representative dimeric interface within the helical structure of the DREP3 CIDE domain. The interface formed by molecules 1 (green) and 2 (yellow) was chosen for analysis. (*b*) Close-up view of the interacting residues in the interface between molecules 1 and 2. The residues involved in the interaction are shown and labelled. Salt bridges formed between one chain and its counterpart are shown as red dashed lines. (*c*) SEC chromatograms of the wild-type (WT) DREP3 CIDE domain and its mutants. The profile obtained from the WT DREP3 CIDE domain is indicated with a black line, that of the D167R mutant with a blue line and that of the K132D mutant with a red line. All SEC experiments were performed using 20 m*M* Tris–HCl pH 7.9, 500 m*M* NaCl buffer. (*d*) SEC-MALS measurement of the D167R mutant showed the absolute molecular mass of the protein. (*e*) CD spectra of the WT DREP3 CIDE domain (black line), the D167R mutant (blue line) and the K132D mutant (red line). The spectra were recorded at 25°C and four scans were performed and averaged using a J-715 spectropolarimeter (Jasco, Oklahoma City, Oklahoma, USA).

The helical assembly of the DREP3 CIDE domain is mediated by repetitive head-to-tail polymerization between oppositely charged interfaces (Figs. 5 ▸ *a* and 5 ▸ *b*). The highly positively charged interface comprising Lys122, Lys132, Lys145 and Lys149 forms a large number of salt bridges and hydrogen bonds with the highly negatively charged interface comprising Asp162, Asp165, Asp167, Asp168 and Glu170 of the neighbouring molecule (Fig. 5 ▸ *b*). This interface buries ∼520 Å^2^ of accessible surface area per molecule. To validate our findings, we performed a mutagenesis analysis. To disrupt the interfaces, the representative interface residues Lys132 for the positively charged interface and Asp167 for the negatively charged interface were mutated to aspartic acid and arginine, respectively, producing K132D and D167R mutants. Based on SEC, the D167R mutant clearly failed to form a helical oligomeric structure, whereas the wild-type DREP3 CIDE domain predominantly formed oligomers in solution (Fig. 5 ▸ *c*). Further analysis of the D167R mutant by SEC-MALS revealed that the molecular weight of the D167R mutant was 10 804 Da (2.4% fitting error), indicating that the D167R mutant existed as a monomer in solution and failed to form a filament-like structure (Fig. 5 ▸ *d*). This means that disruption of the tentative interface detected in the helical filament structure affects the filament assembly of the DREP3 CIDE domain. For the K132D mutant, the expression levels reduced dramatically. Although the effect of the mutation was less clear than that produced by the D167R mutant, the K132D mutation clearly affected the helical assembly of the DREP3 CIDE domain (Fig. 5 ▸ *c*). To ensure that oligomer disruption was not due to structural alterations resulting from mutagenesis, we obtained far-UV CD spectra data. The wild-type and mutant variants showed similar CD spectral patterns typical of mixed α-helix- and β-sheet-containing proteins, with two pronounced minima at 208 and 222 nm, respectively, indicating that the structures were unchanged by mutagenesis (Fig. 1 ▸ *e*).

**Figure 6:**
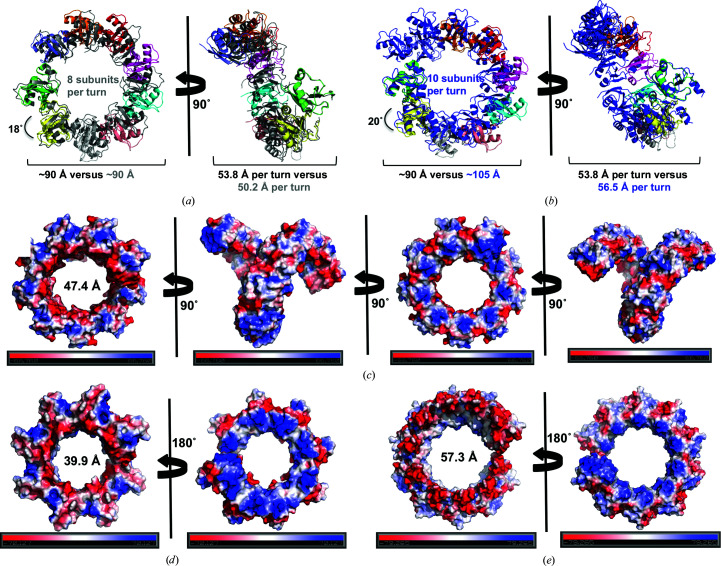
Comparison of the helical filament structure of the DREP3 CIDE domain with those of other CIDE domains. (*a*) Structural alignment of one turn comprised of nine molecules of the DREP3 CIDE domain with one turn comprised of eight molecules of the DREP2 CIDE domain (grey) revealing a conserved helical arrangement. A molecule of the DREP3 CIDE domain adjacent to the aligned molecule needs to rotate 18° to allow superimposition with the corresponding DREP2 CIDE domain. (*b*) Structural alignment of one turn comprised of nine molecules of the DREP3 CIDE domain with one turn comprised of ten molecules of the DREP4 CIDE domain (blue). A molecule of the DREP3 CIDE domain adjacent to the aligned molecule needs to rotate 20° to allow superimposition with the corresponding DREP2 CIDE domain. (*c*) A 360° surface view of the helical filament structure of the DREP3 CIDE domain with displayed electrostatics. The diameter of the hole formed in the helical filament is shown. (*d*, *e*) Electrostatic surface representations of the helical filament structures of the DREP2 (*d*) and DREP4 (*e*) CIDE domains. The diameters of the hole are provided.

Next, we compared the helical filament structure of the DREP3 CIDE domain with the recently revealed helical structures of the DREP2 and DREP4 CIDE domains by superimposition (Choi *et al.*, 2017 ▸; Ha & Park, 2018 ▸). Structural alignment revealed that the neighbouring molecules had an ∼18° rotation for DREP2 and a ∼20° rotation for DREP4 (Figs. 6 ▸ *a* and 6 ▸ *b*). Additionally, nine molecules were involved in one turn of the helical filament in the DREP3 CIDE domain, whereas eight and ten molecules were involved in one turn of the helical filaments of the DREP2 and DREP4 CIDE domains, respectively (Figs. 6 ▸ *a* and 6 ▸ *b*). At ∼105 Å, DREP4 had the largest diameter of the CIDE-domain helical filaments, compared with 90 Å for the DREP2 and DREP3 CIDE domains. Furthermore, the rise of one helical turn was 50.2 Å for DREP2, 53.8 Å for DREP3 and 56.5 Å for DREP4 (Figs. 6 ▸ *a* and 6 ▸ *b*).

The electrostatic surface view of the helical filament structure of the DREP3 CIDE domain showed that the two helical ends carried opposite charges, which extended the filament structure (Fig. 6 ▸ *c*). This helical feature was also observed in the filament structures of the DREP2 and DREP4 CIDE domains (Figs. 6 ▸ *d* and 6 ▸ *e*). Additionally, the diameters of the holes produced by the spiral assemblies of CIDE domains differed, with the largest being produced by the DREP4 domain (∼57.3 Å) and the smallest by the DREP2 CIDE domain (∼39.9 Å) (Figs. 6 ▸ *c*, 6 ▸ *d* and 6 ▸ *e*). These analyses indicated that multiple CIDE domains form helical filament structures in solution, and that these structures vary in length, diameter and helical properties, although all of the domains appear to share a unified mechanism for helical assembly.

## 4. Discussion

The functions of CIDE-domain-containing proteins have expanded from involvement in apoptotic DNA fragmentation as a hallmark of apoptosis to lipid homeostasis and brain synapse functions (Liu *et al.*, 1998 ▸; Sun *et al.*, 2013 ▸; Andlauer *et al.*, 2014 ▸; Traini & Jessup, 2009 ▸). Hetero-oligomeric and homo-oligomeric complex formation via the CIDE domain is critical for the proper functions of CIDE-domain-containing proteins (Otomo *et al.*, 2000 ▸; Choi *et al.*, 2017 ▸). Interestingly, a recent study revealed that the CIDE domain can form a filament-like structure, which is also critical for protein function (Choi *et al.*, 2017 ▸; Ha & Park, 2018 ▸). Additionally, higher-order constructions of macromolecules in cells may be a new trend in signal transduction during cell death and immune function (Wu, 2013 ▸; Xia *et al.*, 2021 ▸; Shi *et al.*, 2020 ▸). For example, the death-domain (DD) superfamily members, including the DD, caspase-recruitment domain, pyrin domain and death-effector domain, also assemble helical filament structures during cellular-signalling events to mediate signal transduction, signal amplification and sometimes proximity-mediated enzyme activation (Park, 2019*a* ▸,*b* ▸; Park *et al.*, 2007 ▸; Xia *et al.*, 2021 ▸). In this regard, CIDE-domain-mediated helical filament formation represents a higher-order structure which is critical for proper apoptotic DNA fragmentation, although it remains uncertain how these structures facilitate lipid homeostasis.

The structure of the DREP3 CIDE domain is another example of a helical filament structure, with an assembly mediated by repetitive head-to-tail polymerization between oppositely charged interfaces. Unlike the more stable and cooperatively formed filament-like structure presented by the DD superfamily (Xia *et al.*, 2021 ▸), but similar to other CIDE domains such as those of DREP4 and DREP2 (Choi *et al.*, 2017 ▸; Kim *et al.*, 2017 ▸; Ha & Park, 2018 ▸), the head-to-tail helical filament observed for the DREP3 CIDE domain was more dynamic in assembly and disassembly. This dynamic property was common in CIDE domains (Kim *et al.*, 2017 ▸). At a high concentration of salt, the filament size of the CIDE domain was dramatically reduced. This is because the main interaction force for CIDE-domain assembly is salt bridges, the strength of which is destroyed by a high concentration of salt. The assembly of the CIDE domain was also affected by the concentration of protein. The CIDE domain formed a longer filament structure at higher protein concentrations. This oppositely charged interaction-mediated head-to-tail filament structure, in which assembly/disassembly are dependent on the salt concentration and the local concentration of protein, might provide a unified mechanism for the CIDE domain. Generally, crystallographic structural studies of open-ended oligomers are challenging due to the formation of endless filaments and occasional heterogeneity in their apparent size. The dynamic assembly of the CIDE domain allowed us to control the size of the filament by adjusting the concentration of protein and salt, which enabled crystal formation and subsequent structural refinement.

Structural comparison of the DREP3 CIDE domain with recently identified helical filament structures of other CIDE domains revealed the diversity of these structures in length, diameter and helical properties, although the CIDE domains share a helical assembly mechanism. Although the function of the CIDE-domain-mediated filament structure in apoptotic DNA fragmentation has been established in a recent structural study (Choi *et al.*, 2017 ▸), its role in lipid metabolism and synapse function require further investigation. In addition, since the CIDE domain forms a dimer in solution, the functional importance of the dimeric form of CIDE domain-containing proteins should be studied and compared with their filament structure. The structure presented here offers novel insights into the helical filament structure formed by the CIDE domain and will aid further investigation of other CIDE-domain-containing proteins to determine their roles in apoptosis, lipid metabolism and brain synapse function.

## Supplementary Material

PDB reference: DREP3, 7v6e

## Figures and Tables

**Table 1:** Data-collection and refinement statistics Values in parentheses are for the outermost resolution shell. ASU, asymmetric unit; r.m.s.d., root-mean-square deviation.

Data collection	
Space group	*P*2_1_2_1_2_1_
*a*, *b*, *c* (Å)	56.46, 125.355, 168.447
α, β, γ (°)	90, 90, 90
Resolution range (Å)	41.95–3.00
Total reflections	224089
Unique reflections	46026
Multiplicity	4.9 (4.7)
Completeness (%)	99.30 (99.34)
Mean *I*/σ(*I*)	13.29 (3.42)
*R*_p.i.m._	0.051 (0.224)
CC_1/2 _	0.987 (0.918)
Wilson *B* factor (Å^2^)	37.2
Refinement	
Resolution range (Å)	41.95–3.00
Reflections	24556 (2406)
*R*_work_ (%)	20.55 (28.51)
*R*_free_ (%)	25.21 (34.73)
No. of molecules in ASU	9
No. of non-H atoms	
Total	5622
Macromolecules	5622
Solvent	0
Average *B* factors (Å^2^)	
Overall	31.67
Macromolecules	31.67
Solvent	0
Ramachandran plot	
Favoured (%)	97.93
Allowed (%)	2.07
Outliers (%)	0.00
Rotamer outliers (%)	0
Clashscore	8.30
R.m.s.d., bond lengths (Å)	0.011
R.m.s.d., angles (°)	1.23
